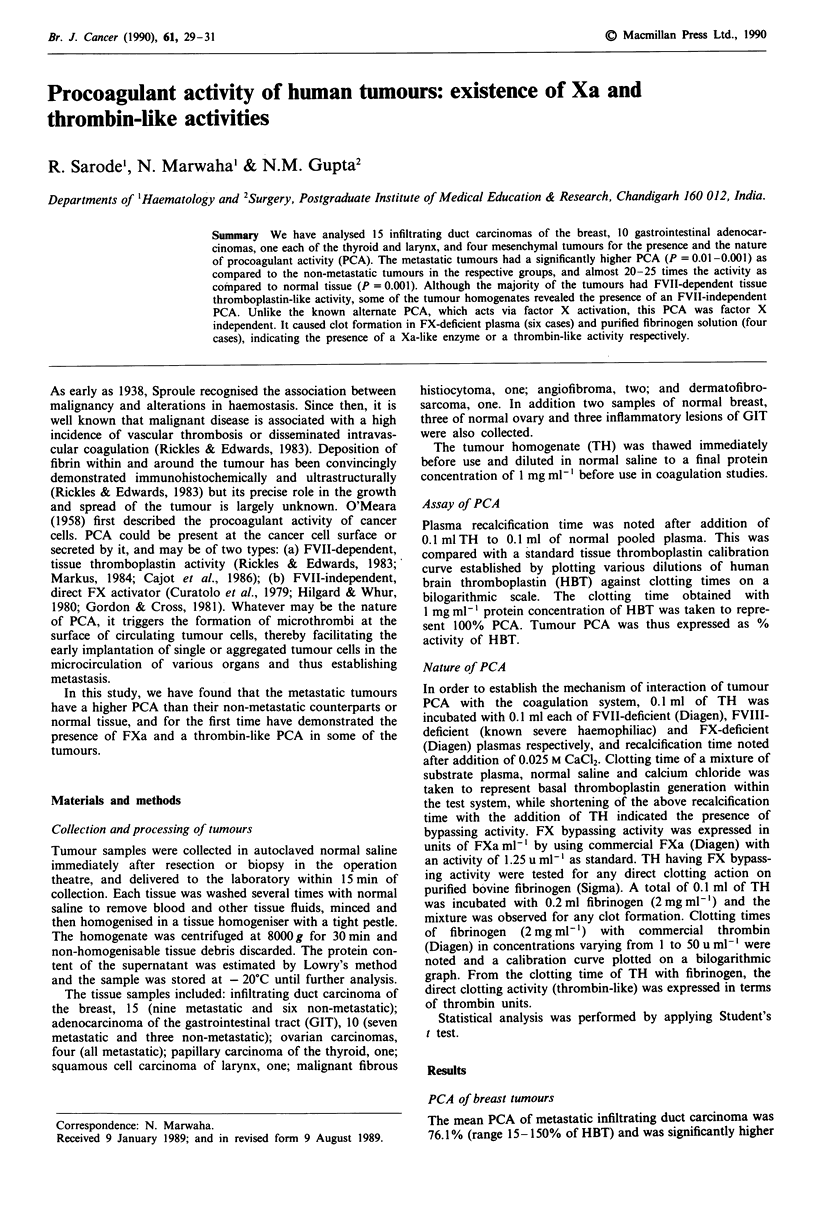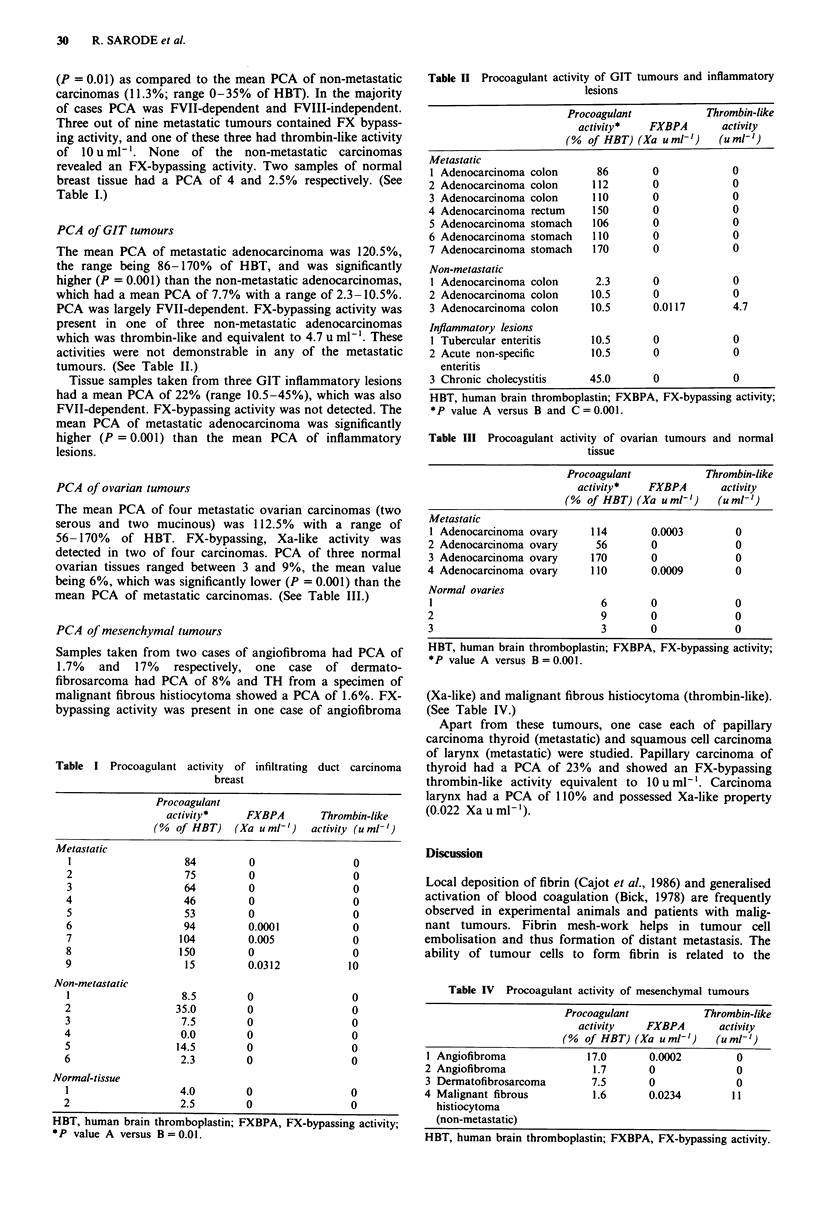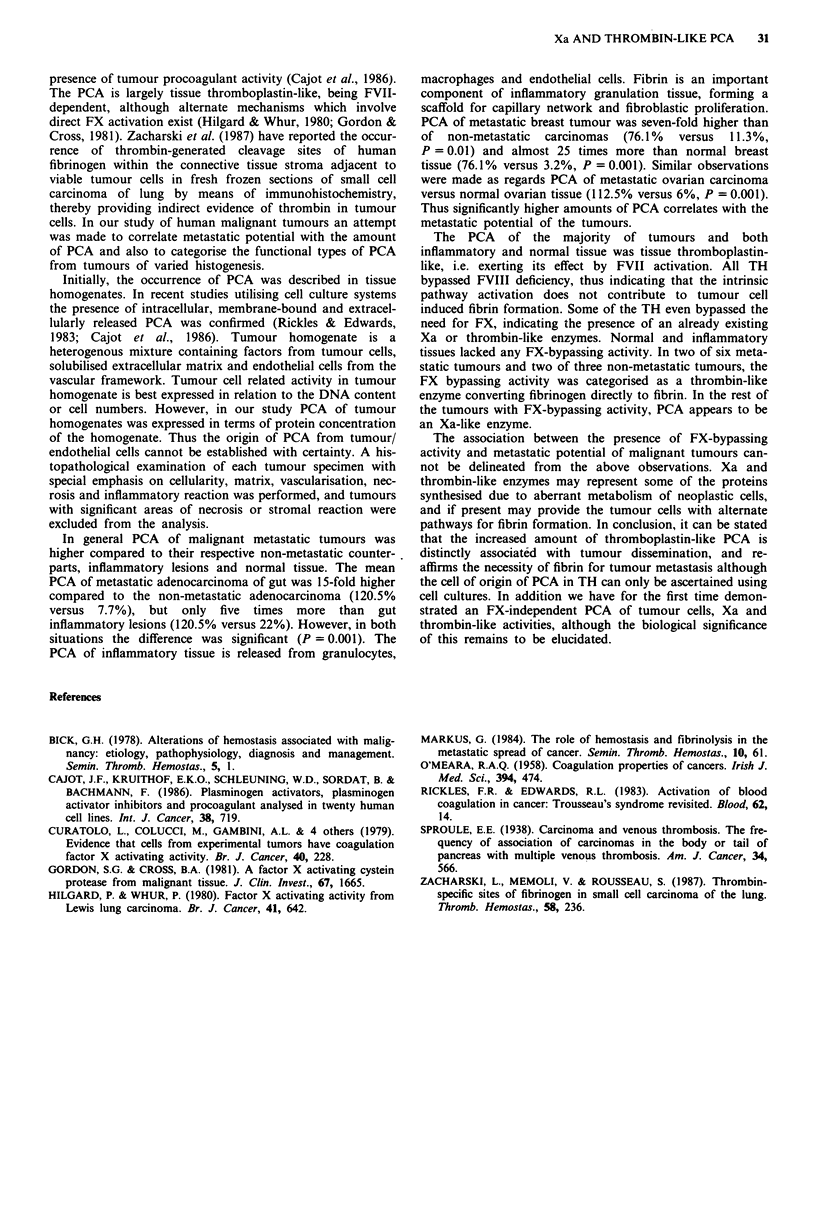# Procoagulant activity of human tumours: existence of Xa and thrombin-like activities.

**DOI:** 10.1038/bjc.1990.7

**Published:** 1990-01

**Authors:** R. Sarode, N. Marwaha, N. M. Gupta

**Affiliations:** Department of Haematology, Postgraduate Institute of Medical Education & Research, Chandigarh, India.

## Abstract

We have analysed 15 infiltrating duct carcinomas of the breast, 10 gastrointestinal adenocarcinomas, one each of the thyroid and larynx, and four mesenchymal tumours for the presence and the nature of procoagulant activity (PCA). The metastatic tumours had a significantly higher PCA (P = 0.01-0.001) as compared to the non-metastatic tumours in the respective groups, and almost 20-25 times the activity as compared to normal tissue (P = 0.001). Although the majority of the tumours had FVII-dependent tissue thromboplastin-like activity, some of the tumour homogenates revealed the presence of an FVII-independent PCA. Unlike the known alternate PCA, which acts via factor X activation, this PCA was factor X independent. It caused clot formation in FX-deficient plasma (six cases) and purified fibrinogen solution (four cases), indicating the presence of a Xa-like enzyme or a thrombin-like activity respectively.


*Br. J. Cancer* (1990), **61**, 29–31 © Macmillan Press Ltd., 1990

### Procoagulant activity of human tumours: existence of Xa and thrombin-like activities

R. Sarode[1], N. Marwaha[1] & N.M. Gupta[2]

*Departments of [1]Haematology and [2]Surgery, Postgraduate Institute of Medical Education & Research, Chandigarh 160 012, India.*

**Summary** We have analysed 15 infiltrating duct carcinomas of the breast, 10 gastrointestinal adenocarcinomas, one each of the thyroid and larynx, and four mesenchymal tumours for the presence and the nature of procoagulant activity (PCA). The metastatic tumours had a significantly higher PCA ($P = 0.01-0.001$) as compared to the non-metastatic tumours in the respective groups, and almost 20–25 times the activity as compared to normal tissue ($P = 0.001$). Although the majority of the tumours had FVII-dependent tissue thromboplastin-like activity, some of the tumour homogenates revealed the presence of an FVII-independent PCA. Unlike the known alternate PCA, which acts via factor X activation, this PCA was factor X independent. It caused clot formation in FX-deficient plasma (six cases) and purified fibrinogen solution (four cases), indicating the presence of a Xa-like enzyme or a thrombin-like activity respectively.

As early as 1938, Sproule recognised the association between malignancy and alterations in haemostasis. Since then, it is well known that malignant disease is associated with a high incidence of vascular thrombosis or disseminated intravascular coagulation (Rickles & Edwards, 1983). Deposition of fibrin within and around the tumour has been convincingly demonstrated immunohistochemically and ultrastructurally (Rickles & Edwards, 1983) but its precise role in the growth and spread of the tumour is largely unknown. O'Meara (1958) first described the procoagulant activity of cancer cells. PCA could be present at the cancer cell surface or secreted by it, and may be of two types: (a) FVII-dependent, tissue thromboplastin activity (Rickles & Edwards, 1983; Markus, 1984; Cajot *et al.*, 1986); (b) FVII-independent, direct FX activator (Curatolo *et al.*, 1979; Hilgard & Whur, 1980; Gordon & Cross, 1981). Whatever may be the nature of PCA, it triggers the formation of microthrombi at the surface of circulating tumour cells, thereby facilitating the early implantation of single or aggregated tumour cells in the microcirculation of various organs and thus establishing metastasis.

In this study, we have found that the metastatic tumours have a higher PCA than their non-metastatic counterparts or normal tissue, and for the first time have demonstrated the presence of FXa and a thrombin-like PCA in some of the tumours.

### Materials and methods

### *Collection and processing of tumours*

Tumour samples were collected in autoclaved normal saline immediately after resection or biopsy in the operation theatre, and delivered to the laboratory within 15 min of collection. Each tissue was washed several times with normal saline to remove blood and other tissue fluids, minced and then homogenised in a tissue homogeniser with a tight pestle. The homogenate was centrifuged at 8000 *g* for 30 min and non-homogenisable tissue debris discarded. The protein content of the supernatant was estimated by Lowry's method and the sample was stored at − 20°C until further analysis.

The tissue samples included: infiltrating duct carcinoma of the breast, 15 (nine metastatic and six non-metastatic); adenocarcinoma of the gastrointestinal tract (GIT), 10 (seven metastatic and three non-metastatic); ovarian carcinomas, four (all metastatic); papillary carcinoma of the thyroid, one; squamous cell carcinoma of larynx, one; malignant fibrous histiocytoma, one; angiofibroma, two; and dermatofibrosarcoma, one. In addition two samples of normal breast, three of normal ovary and three inflammatory lesions of GIT were also collected.

The tumour homogenate (TH) was thawed immediately before use and diluted in normal saline to a final protein concentration of 1 mg $ml^{-1}$ before use in coagulation studies.

### *Assay of PCA*

Plasma recalcification time was noted after addition of 0.1 ml TH to 0.1 ml of normal pooled plasma. This was compared with a standard tissue thromboplastin calibration curve established by plotting various dilutions of human brain thromboplastin (HBT) against clotting times on a bilogarithmic scale. The clotting time obtained with 1 mg $ml^{-1}$ protein concentration of HBT was taken to represent 100% PCA. Tumour PCA was thus expressed as % activity of HBT.

### *Nature of PCA*

In order to establish the mechanism of interaction of tumour PCA with the coagulation system, 0.1 ml of TH was incubated with 0.1 ml each of FVII-deficient (Diagen), FVIII-deficient (known severe haemophiliac) and FX-deficient (Diagen) plasmas respectively, and recalcification time noted after addition of 0.025 M $CaCl_2$. Clotting time of a mixture of substrate plasma, normal saline and calcium chloride was taken to represent basal thromboplastin generation within the test system, while shortening of the above recalcification time with the addition of TH indicated the presence of bypassing activity. FX bypassing activity was expressed in units of FXa $ml^{-1}$ by using commercial FXa (Diagen) with an activity of 1.25 u $ml^{-1}$ as standard. TH having FX bypassing activity were tested for any direct clotting action on purified bovine fibrinogen (Sigma). A total of 0.1 ml of TH was incubated with 0.2 ml fibrinogen (2 mg $ml^{-1}$) and the mixture was observed for any clot formation. Clotting times of fibrinogen (2 mg $ml^{-1}$) with commercial thrombin (Diagen) in concentrations varying from 1 to 50 u $ml^{-1}$ were noted and a calibration curve plotted on a bilogarithmic graph. From the clotting time of TH with fibrinogen, the direct clotting activity (thrombin-like) was expressed in terms of thrombin units.

Statistical analysis was performed by applying Student's *t* test.

### Results

### *PCA of breast tumours*

The mean PCA of metastatic infiltrating duct carcinoma was 76.1% (range 15–150% of HBT) and was significantly higher

Correspondence: N. Marwaha.
Received 9 January 1989; and in revised form 9 August 1989.

($P = 0.01$) as compared to the mean PCA of non-metastatic carcinomas (11.3%; range 0–35% of HBT). In the majority of cases PCA was FVII-dependent and FVIII-independent. Three out of nine metastatic tumours contained FX bypassing activity, and one of these three had thrombin-like activity of 10 u $ml^{-1}$. None of the non-metastatic carcinomas revealed an FX-bypassing activity. Two samples of normal breast tissue had a PCA of 4 and 2.5% respectively. (See Table I.)

### PCA of GIT tumours

The mean PCA of metastatic adenocarcinoma was 120.5%, the range being 86–170% of HBT, and was significantly higher ($P = 0.001$) than the non-metastatic adenocarcinomas, which had a mean PCA of 7.7% with a range of 2.3–10.5%. PCA was largely FVII-dependent. FX-bypassing activity was present in one of three non-metastatic adenocarcinomas which was thrombin-like and equivalent to 4.7 u $ml^{-1}$. These activities were not demonstrable in any of the metastatic tumours. (See Table II.)

Tissue samples taken from three GIT inflammatory lesions had a mean PCA of 22% (range 10.5–45%), which was also FVII-dependent. FX-bypassing activity was not detected. The mean PCA of metastatic adenocarcinoma was significantly higher ($P = 0.001$) than the mean PCA of inflammatory lesions.

### PCA of ovarian tumours

The mean PCA of four metastatic ovarian carcinomas (two serous and two mucinous) was 112.5% with a range of 56–170% of HBT. FX-bypassing, Xa-like activity was detected in two of four carcinomas. PCA of three normal ovarian tissues ranged between 3 and 9%, the mean value being 6%, which was significantly lower ($P = 0.001$) than the mean PCA of metastatic carcinomas. (See Table III.)

### PCA of mesenchymal tumours

Samples taken from two cases of angiofibroma had PCA of 1.7% and 17% respectively, one case of dermatofibrosarcoma had PCA of 8% and TH from a specimen of malignant fibrous histiocytoma showed a PCA of 1.6%. FX-bypassing activity was present in one case of angiofibroma (Xa-like) and malignant fibrous histiocytoma (thrombin-like). (See Table IV.)

Apart from these tumours, one case each of papillary carcinoma thyroid (metastatic) and squamous cell carcinoma of larynx (metastatic) were studied. Papillary carcinoma of thyroid had a PCA of 23% and showed an FX-bypassing thrombin-like activity equivalent to 10 u $ml^{-1}$. Carcinoma larynx had a PCA of 110% and possessed Xa-like property (0.022 Xa u $ml^{-1}$).

**Table I** Procoagulant activity of infiltrating duct carcinoma breast

| | *Procoagulant activity* (% of HBT)* | *FXBPA (Xa u $ml^{-1}$)* | *Thrombin-like activity (u $ml^{-1}$)* |
|---|---|---|---|
| *Metastatic* | | | |
| 1 | 84 | 0 | 0 |
| 2 | 75 | 0 | 0 |
| 3 | 64 | 0 | 0 |
| 4 | 46 | 0 | 0 |
| 5 | 53 | 0 | 0 |
| 6 | 94 | 0.0001 | 0 |
| 7 | 104 | 0.005 | 0 |
| 8 | 150 | 0 | 0 |
| 9 | 15 | 0.0312 | 10 |
| *Non-metastatic* | | | |
| 1 | 8.5 | 0 | 0 |
| 2 | 35.0 | 0 | 0 |
| 3 | 7.5 | 0 | 0 |
| 4 | 0.0 | 0 | 0 |
| 5 | 14.5 | 0 | 0 |
| 6 | 2.3 | 0 | 0 |
| *Normal-tissue* | | | |
| 1 | 4.0 | 0 | 0 |
| 2 | 2.5 | 0 | 0 |

HBT, human brain thromboplastin; FXBPA, FX-bypassing activity; *$P$ value A versus B = 0.01.

**Table II** Procoagulant activity of GIT tumours and inflammatory lesions

| | *Procoagulant activity* (% of HBT)* | *FXBPA (Xa u $ml^{-1}$)* | *Thrombin-like activity (u $ml^{-1}$)* |
|---|---|---|---|
| *Metastatic* | | | |
| 1 Adenocarcinoma colon | 86 | 0 | 0 |
| 2 Adenocarcinoma colon | 112 | 0 | 0 |
| 3 Adenocarcinoma colon | 110 | 0 | 0 |
| 4 Adenocarcinoma rectum | 150 | 0 | 0 |
| 5 Adenocarcinoma stomach | 106 | 0 | 0 |
| 6 Adenocarcinoma stomach | 110 | 0 | 0 |
| 7 Adenocarcinoma stomach | 170 | 0 | 0 |
| *Non-metastatic* | | | |
| 1 Adenocarcinoma colon | 2.3 | 0 | 0 |
| 2 Adenocarcinoma colon | 10.5 | 0 | 0 |
| 3 Adenocarcinoma colon | 10.5 | 0.0117 | 4.7 |
| *Inflammatory lesions* | | | |
| 1 Tubercular enteritis | 10.5 | 0 | 0 |
| 2 Acute non-specific enteritis | 10.5 | 0 | 0 |
| 3 Chronic cholecystitis | 45.0 | 0 | 0 |

HBT, human brain thromboplastin; FXBPA, FX-bypassing activity; *$P$ value A versus B and C = 0.001.

**Table III** Procoagulant activity of ovarian tumours and normal tissue

| | *Procoagulant activity* (% of HBT)* | *FXBPA (Xa u $ml^{-1}$)* | *Thrombin-like activity (u $ml^{-1}$)* |
|---|---|---|---|
| *Metastatic* | | | |
| 1 Adenocarcinoma ovary | 114 | 0.0003 | 0 |
| 2 Adenocarcinoma ovary | 56 | 0 | 0 |
| 3 Adenocarcinoma ovary | 170 | 0 | 0 |
| 4 Adenocarcinoma ovary | 110 | 0.0009 | 0 |
| *Normal ovaries* | | | |
| 1 | 6 | 0 | 0 |
| 2 | 9 | 0 | 0 |
| 3 | 3 | 0 | 0 |

HBT, human brain thromboplastin; FXBPA, FX-bypassing activity; *$P$ value A versus B = 0.001.

### Discussion

Local deposition of fibrin (Cajot et al., 1986) and generalised activation of blood coagulation (Bick, 1978) are frequently observed in experimental animals and patients with malignant tumours. Fibrin mesh-work helps in tumour cell embolisation and thus formation of distant metastasis. The ability of tumour cells to form fibrin is related to the

**Table IV** Procoagulant activity of mesenchymal tumours

| | *Procoagulant activity (% of HBT)* | *FXBPA (Xa u $ml^{-1}$)* | *Thrombin-like activity (u $ml^{-1}$)* |
|---|---|---|---|
| 1 Angiofibroma | 17.0 | 0.0002 | 0 |
| 2 Angiofibroma | 1.7 | 0 | 0 |
| 3 Dermatofibrosarcoma | 7.5 | 0 | 0 |
| 4 Malignant fibrous histiocytoma (non-metastatic) | 1.6 | 0.0234 | 11 |

HBT, human brain thromboplastin; FXBPA, FX-bypassing activity.

presence of tumour procoagulant activity (Cajot *et al.*, 1986). The PCA is largely tissue thromboplastin-like, being FVII-dependent, although alternate mechanisms which involve direct FX activation exist (Hilgard & Whur, 1980; Gordon & Cross, 1981). Zacharski *et al.* (1987) have reported the occurrence of thrombin-generated cleavage sites of human fibrinogen within the connective tissue stroma adjacent to viable tumour cells in fresh frozen sections of small cell carcinoma of lung by means of immunohistochemistry, thereby providing indirect evidence of thrombin in tumour cells. In our study of human malignant tumours an attempt was made to correlate metastatic potential with the amount of PCA and also to categorise the functional types of PCA from tumours of varied histogenesis.

Initially, the occurrence of PCA was described in tissue homogenates. In recent studies utilising cell culture systems the presence of intracellular, membrane-bound and extracellularly released PCA was confirmed (Rickles & Edwards, 1983; Cajot *et al.*, 1986). Tumour homogenate is a heterogenous mixture containing factors from tumour cells, solubilised extracellular matrix and endothelial cells from the vascular framework. Tumour cell related activity in tumour homogenate is best expressed in relation to the DNA content or cell numbers. However, in our study PCA of tumour homogenates was expressed in terms of protein concentration of the homogenate. Thus the origin of PCA from tumour/endothelial cells cannot be established with certainty. A histopathological examination of each tumour specimen with special emphasis on cellularity, matrix, vascularisation, necrosis and inflammatory reaction was performed, and tumours with significant areas of necrosis or stromal reaction were excluded from the analysis.

In general PCA of malignant metastatic tumours was higher compared to their respective non-metastatic counterparts, inflammatory lesions and normal tissue. The mean PCA of metastatic adenocarcinoma of gut was 15-fold higher compared to the non-metastatic adenocarcinoma (120.5% versus 7.7%), but only five times more than gut inflammatory lesions (120.5% versus 22%). However, in both situations the difference was significant ($P = 0.001$). The PCA of inflammatory tissue is released from granulocytes, macrophages and endothelial cells. Fibrin is an important component of inflammatory granulation tissue, forming a scaffold for capillary network and fibroblastic proliferation. PCA of metastatic breast tumour was seven-fold higher than of non-metastatic carcinomas (76.1% versus 11.3%, $P = 0.01$) and almost 25 times more than normal breast tissue (76.1% versus 3.2%, $P = 0.001$). Similar observations were made as regards PCA of metastatic ovarian carcinoma versus normal ovarian tissue (112.5% versus 6%, $P = 0.001$). Thus significantly higher amounts of PCA correlates with the metastatic potential of the tumours.

The PCA of the majority of tumours and both inflammatory and normal tissue was tissue thromboplastin-like, i.e. exerting its effect by FVII activation. All TH bypassed FVIII deficiency, thus indicating that the intrinsic pathway activation does not contribute to tumour cell induced fibrin formation. Some of the TH even bypassed the need for FX, indicating the presence of an already existing Xa or thrombin-like enzymes. Normal and inflammatory tissues lacked any FX-bypassing activity. In two of six metastatic tumours and two of three non-metastatic tumours, the FX bypassing activity was categorised as a thrombin-like enzyme converting fibrinogen directly to fibrin. In the rest of the tumours with FX-bypassing activity, PCA appears to be an Xa-like enzyme.

The association between the presence of FX-bypassing activity and metastatic potential of malignant tumours cannot be delineated from the above observations. Xa and thrombin-like enzymes may represent some of the proteins synthesised due to aberrant metabolism of neoplastic cells, and if present may provide the tumour cells with alternate pathways for fibrin formation. In conclusion, it can be stated that the increased amount of thromboplastin-like PCA is distinctly associated with tumour dissemination, and reaffirms the necessity of fibrin for tumour metastasis although the cell of origin of PCA in TH can only be ascertained using cell cultures. In addition we have for the first time demonstrated an FX-independent PCA of tumour cells, Xa and thrombin-like activities, although the biological significance of this remains to be elucidated.

**References**

BICK, G.H. (1978). Alterations of hemostasis associated with malignancy: etiology, pathophysiology, diagnosis and management. *Semin. Thromb. Hemostas.*, **5**, 1.

CAJOT, J.F., KRUITHOF, E.K.O., SCHLEUNING, W.D., SORDAT, B. & BACHMANN, F. (1986). Plasminogen activators, plasminogen activator inhibitors and procoagulant analysed in twenty human cell lines. *Int. J. Cancer*, **38**, 719.

CURATOLO, L., COLUCCI, M., GAMBINI, A.L. & 4 others (1979). Evidence that cells from experimental tumors have coagulation factor X activating activity. *Br. J. Cancer*, **40**, 228.

GORDON, S.G. & CROSS, B.A. (1981). A factor X activating cystein protease from malignant tissue. *J. Clin. Invest.*, **67**, 1665.

HILGARD, P. & WHUR, P. (1980). Factor X activating activity from Lewis lung carcinoma. *Br. J. Cancer*, **41**, 642.

MARKUS, G. (1984). The role of hemostasis and fibrinolysis in the metastatic spread of cancer. *Semin. Thromb. Hemostas.*, **10**, 61.

O'MEARA, R.A.Q. (1958). Coagulation properties of cancers. *Irish J. Med. Sci.*, **394**, 474.

RICKLES, F.R. & EDWARDS, R.L. (1983). Activation of blood coagulation in cancer: Trousseau's syndrome revisited. *Blood*, **62**, 14.

SPROULE, E.E. (1938). Carcinoma and venous thrombosis. The frequency of association of carcinomas in the body or tail of pancreas with multiple venous thrombosis. *Am. J. Cancer*, **34**, 566.

ZACHARSKI, L., MEMOLI, V. & ROUSSEAU, S. (1987). Thrombin-specific sites of fibrinogen in small cell carcinoma of the lung. *Thromb. Hemostas.*, **58**, 236.